# Hypoglycemic and Hypolipidemic Effects of Ethanolic Extract of *Mirabilis jalapa* L. Root on Normal and Diabetic Mice

**DOI:** 10.1155/2012/257374

**Published:** 2012-02-27

**Authors:** Ji-Yin Zhou, Shi-Wen Zhou, Sheng-Ya Zeng, Jian-Yun Zhou, Ming-Jin Jiang, Yan He

**Affiliations:** ^1^Base for Drug Clinical Trial, Xinqiao Hospital, Third Military Medical University, Chongqing 400037, China; ^2^Research Division, Xinqiao Hospital, Third Military Medical University, Chongqing 400037, China

## Abstract

The present study investigated the insulin sensitivity, hypoglycemic, and hypolipidemic activities of ethanolic extract of *Mirabilis jalapa* L. root (EEM) in normal and diabetic mice. After induction of diabetes with streptozotocin, both normal and diabetic mice were singly or repeatedly for 28 days administrated with EEM at doses of 2, 4, 8 g/kg, respectively. Before induction of diabetes, mice were administrated with EEM at doses of 2, 4, 8 g/kg for 14 days and were injected with streptozotocin and continued on EEM administration for another 28 days. Both after and before induction of diabetes, repeated administration with 4, 8 g/kg EEM continually lowered blood glucose level, decreased serum insulin level and improved insulin sensitivity index, and lowered serum total cholesterol, triglyceride levels and triglyceride content in liver and skeletal muscle, and increased glycogen content in these tissues; but repeated administration had no influence on those indexes of normal mice. Single administration with EEM (4, 8 g/kg) showed hypoglycemic effect in oral glucose tolerance test in normal and diabetic mice. Single administration with EEM had no hypoglycemic and hypolipidemic effects on normal and diabetic mice. These results suggest that EEM possesses both potential insulin sensitivity, hypoglycemic, and hypolipidemic effects on diabetes.

## 1. Introduction

Type 2 diabetes mellitus, a metabolic disorder with manifestations of hyperglycemia and hyperinsulinemia, is one of the commonest chronic diseases worldwide [1]. Though different types of oral hypoglycemic agents are available for the treatment of diabetes mellitus, there is increasing demand by patients to use antidiabetic natural products because of the undesirable side effects of the existing drugs. In many countries, much attention has been paid to find novel type of natural antidiabetic drugs from various medicinal plants [2–5]. With thousands of years of medical practice, a great deal of valuable experience has been accumulated in traditional Chinese medical system for diabetic therapy and several herbs have been used to improve the hyperglycemic condition in diabetic patients. Because of their effectiveness, limited side effects, and relatively low cost, herbal drugs are widely prescribed even when their biologically active compounds are unknown [6].

*Mirabilis jalapa* L. belongs to the family Nyctaginaceae and is known as “four o'clock,” “maravilha,” “belle de nuit,” “buenas tardes,” “dondiego de noche,” “jalap,” “noche buena,” or “Tzu Mo Li” in different areas. *Mirabilis jalapa* L. is widely used to treat dysentery, diarrhea, muscular pain, and abdominal colics in different countries [7–9], and its extract has antibacterial, antiviral, and antifungal functions [10–12]. In China, *Mirabilis jalapa *L. is widely distributed and commonly used with its root and has been used as traditional Chinese medicine and ethnic drug to treat diabetes [13, 14], constipation [15], genitourinary system disorders, and injuries [16]. *Mirabilis jalapa *L. root contains alkaloids, glycosides, carbohydrates, and phytosterols by phytochemical analysis [9, 17]. According to literatures, trigonelline is one of the components of *Mirabilis jalapa* L. root [18]. Trigonelline has been shown to reduce blood glucose concentrations in rats [19, 20] and in human [21, 22]. Only recently, Piyali et al. [17] reported the hypoglycemic and hypolipidemic effects of *Mirabilis jalapa* L. root on streptozotocin-induced diabetic Wistar albino rats.

In this study, the experiments were designed to detect the insulin sensitivity and hypoglycemic and hypolipidemic activities of ethanolic extract of *Mirabilis jalapa *L. root (EEM) on both diabetic and normal mice after induction with streptozotocin with both singly and repeatedly administration, and on diabetes before induction with repeatedly administration, and compared with glibenclamide as a reference standard. Oral glucose tolerance test was also measured on normal and diabetic mice after single EEM administration.

## 2. Materials and Methods

### 2.1. Plant Material and EEM Preparation

The root of *Mirabilis jalapa* L. was collected from Chongqing city of China. They were taxonomically authenticated by Dr. Guo-yue Zhong, and a voucher specimen of the plant (TRI7374) has been deposited in Chongqing Academy of Chinese Materia Medica.

The root was dried in shade and powdered in a grinder. For the preparation of EEM, dried powder of *Mirabilis jalapa* L. root (2 kg) was refluxed with 10 volumes of 70% ethanol three times, one hour for each time. The combined ethanolic extract was filtered using a Millipore filter (Millipore 0.2 mm, St. Quentin en Yvelines, France) to remove particulate matter. The ethanolic extract was concentrated and dried into solid residues in vacuo (95.08 mg extract/g crude material). The residue was stored at 4°C, and the desired dose (g of *Mirabilis jalapa* L. root of per kg body weight) was reconstituted in distilled water daily, just before administration.

*Mirabilis jalapa* L. root has recently been studied for its chemical composition by Piyali et al. [17]. The aqueous extract had the highest (3.09%) extractive value, and the ethyl acetate extract had the lowest (0.13%) extractive value. The petroleum ether extract contained alkaloids, and benzene and chloroform extracts both contained glycosides, phytosterols; acetone extract contained alkaloids, carbohydrates; ethyl acetate extract contained alkaloid, carbohydrates, and glycoside; methanol, ethanol, and water extracts all contained alkaloids, carbohydrates, and phytosterols.

### 2.2. Preliminary Phytochemical Analysis

High performance liquid chromatography method has been developed for the analysis of trigonelline in *Mirabilis jalapa* L. root [18]. High-performance liquid chromatography analysis was performed on a YWG-C18 column (250 × 4.6 mm, 10 *μ*m); mobile phases: acetonitrile water (80 : 20, v/v); flow rate: 0.8 mL/min. Peaks were analyzed spectroscopically at 265 nm with a UV-visible-light detector. The EEM solutions were quantified by spiking with a known amount of standard (trigonelline) and also by comparing the area under curve. The repeatability of the method was evaluated by injecting the solution of EEM and standard solution for three times, and the relative standard deviation percentage was calculated.

### 2.3. Animals and Drugs

 Male KM mice (18–22 g) were purchased from Third Military Medical University, China. The animals were given a standard pellet diet and water ad libitum and were maintained under standard environmental conditions (20 ± 2°C, 12 h of light/dark cycle). Streptozotocin was purchased from Sigma Co. Ltd, St. Louis, MO, USA. Glibenclamide (no. 20100224, purity 99.56%) and metformin hydrochloride (no. 20110809, purity 99.60%) were purchased from Wuhan Yuancheng Technology Development Co. Ltd, China. Insulin radioimmunoassay kit was provided by Beijing North Institute of Biological Technology, China. All other chemicals were obtained from local sources and were analytical grade. Throughout the test, experimental mice were processed in accordance with the UK Animals (Scientific Procedures) Act 1986 and associated guidelines.

### 2.4. Study of EEM on Normal Mice

 Sixteen-hour-fasted normal mice were both randomly assigned to five different groups (*n* = 10 in each group). The normal control group received distilled water, and normal treated groups received glibenclamide at a dose of 0.76 mg/kg and EEM at doses of 2, 4, and 8 g/kg, respectively. The selected doses of EEM were according to the literature [13].

Single administration procedure is as follows: the drug solutions or distilled water were administered orally 0.2 mL/10 g body weight by gastric intubation using a syringe once daily at 9:00 a.m. Two hours after a single administration, mice were anaesthetized, and the eye socket blood of mouse was collected for measurement of blood glucose level with a portable glucometer (OneTouch SureStep Meter, LifeScan Company, USA), serum insulin, and total cholesterol (TC) and triglyceride (TG) levels. The liver and skeletal muscle were removed and immediately frozen in liquid nitrogen.

The repeated administration procedure is as follows: mice were treated as single administration once daily for 28 days. Body weights were weighed every two weeks. At 14th day, 16-hour-fasted blood sample was collected from the tail for blood glucose measurement. At 28th day, mice were anaesthetized and 16-hour-fasted eye socket blood of mouse was collected for measurement of blood glucose, serum insulin, and TC and TG levels. The liver and skeletal muscle were also collected and frozen in liquid nitrogen.

### 2.5. Study of EEM on Diabetic Mice

The animals were fasted for 16 h prior to the induction of diabetes. Streptozotocin was freshly prepared in 0.1 mol/L citrate buffer solution (pH4.5) and was intraperitoneally injected to mice with a single dose of 60 mg/kg. After three days, 16-hour-fasted blood sample was collected from the tail for measurement of fasting blood glucose level. Mice with fasting blood glucose level higher than 11.1 mmol/L were considered diabetic ones and were randomly divided into five different groups (*n* = 10 in each group). The diabetic control group received distilled water, and diabetic treated groups received glibenclamide at a dose of 0.76 mg/kg and EEM at doses of 2, 4, and 8 g/kg, respectively. The single and repeated administration procedures were the same as in Subsection 2.4.

### 2.6. Oral Glucose Tolerance Test

 Prior to an oral glucose tolerance test, both normal and diabetic mice induced by the above-mentioned method with fasting blood glucose level higher than 11.1 mmol/L were fasted for 16 h. Distilled water (control), two reference drugs, glibenclamide (2.5 mg/kg) and metformin (50 mg/kg), and three doses of EEM (2, 4, and 8 g/kg) were orally administered to groups of 10 mice each. Two hours later, glucose (2.0 g/kg) was orally administrated to each mouse. Blood samples were taken from tail vein at 0 (just before the glucose administration), 15, 30, 60, and 120 min for the assay of glucose.

### 2.7. Measurement of Insulin Level in Serum

 Serum insulin concentration was measured by commercial kits according to the manufacturer's instructions. Insulin sensitivity index (ISI) = log (1/fasting plasma glucose × serum insulin).

### 2.8. Measurement of TC and TG Levels in Blood

TC and TG levels were determined by spectrophotometry with commercial kit provided by Nanjing Jiancheng Bioengineering Institute, China.

### 2.9. Measurement of Glycogen and TG Contents in Liver and Skeletal Muscle

Glycogen content was determined by spectrophotometry with commercial kit provided by Nanjing Jiancheng Bioengineering Institute, China. TG content in liver and skeletal muscle was measured by the method of Hu et al. [23]. The frozen liver (3–5 mg) and skeletal muscle (15–20 mg) were used for TG extraction. Each frozen tissue was added to 0.3 mL heptane-isopropanol-tween mixture (3 : 2 : 0.01 by volume) and homogenized. This homogenate was centrifuged at 1500 × *g* at 4°C for 15 min. Supernatants (upper phase contained extracted TG) were collected and evaporated with vacuum centrifuge. The TG content was determined by spectrophotometry with commercial kit (Nanjing Jiancheng Bioengineering Institute, China).

### 2.10. Statistical Analyses

All data were expressed as mean ± SD. Statistical analysis was performed with one-way ANOVA followed by Tukey post hoc test for multiple comparisons. *P* < 0.05 was considered significant.

## 3. Results

### 3.1. Phytochemical Study of EEM

**Figure 1:**
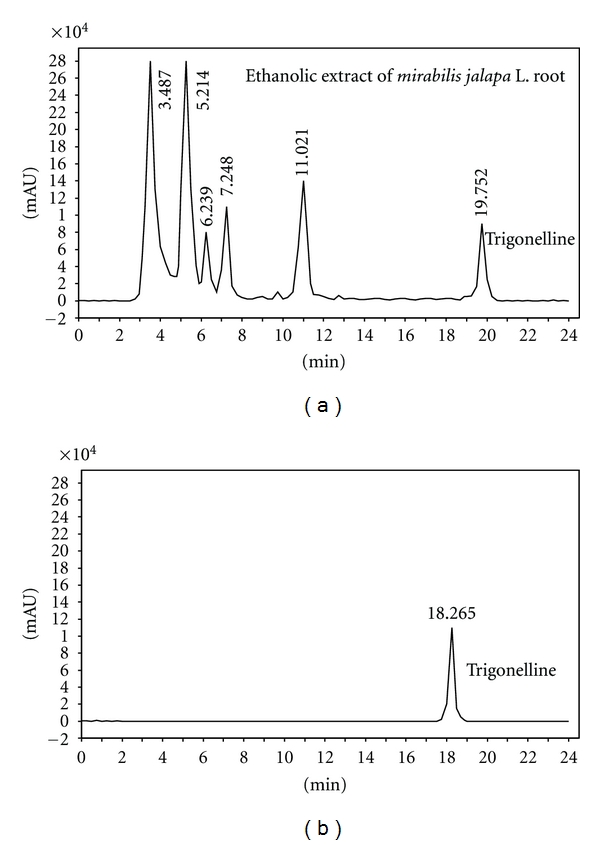
Representative high-performance liquid chromatography chromatograms of EEM and trigonelline (*R*_*t*_ = 19.725).

Phytochemical study of EEM confirmed the presence of trigonelline. The content and variety of trigonelline which has a maximum absorbance at 256 nm is 0.1818 ± 0.0109 mg/g *Mirabilis jalapa* L. root (Figure 1).

### 3.2. Effects of EEM on Blood Glucose Level of Single Administration

**Figure 2:**
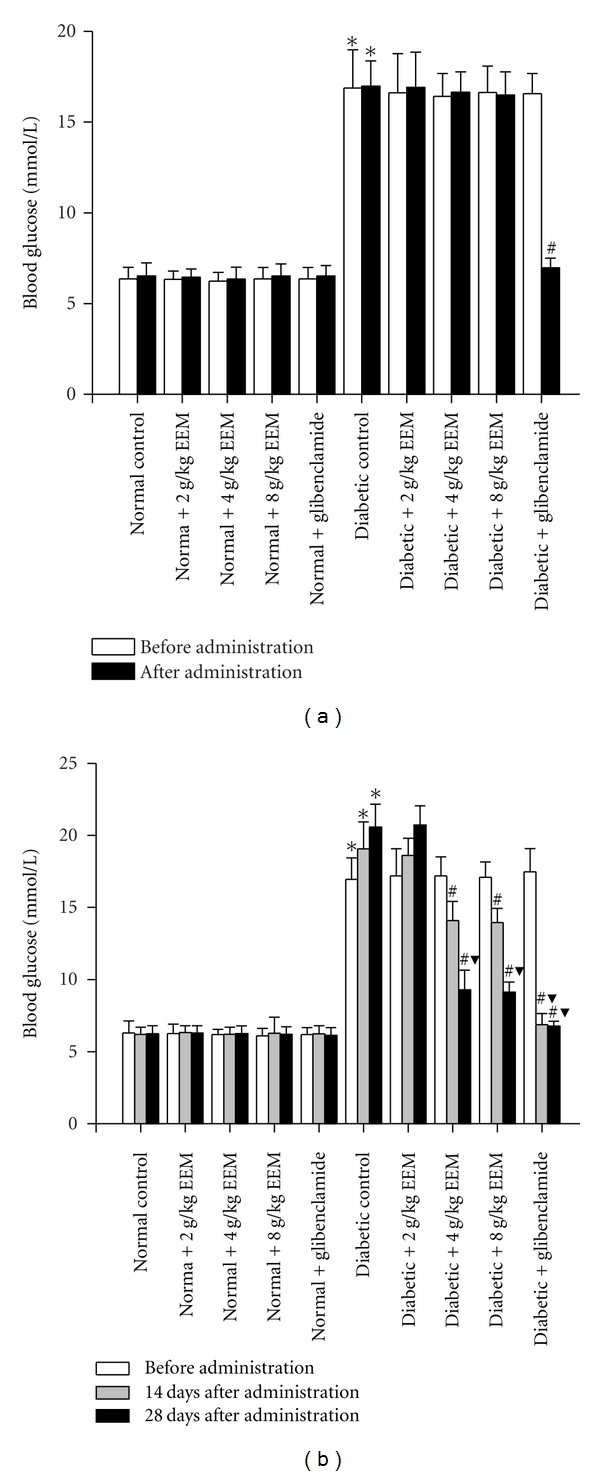
Effects of single and repeated EEM administration on blood glucose level in normal and diabetic mice. Data are expressed as mean ± SD, *n* = 10 mice per group. *P < 0.01, compared to normal control. ^#^ *P* < 0.01, compared to diabetic control. ^*▼*^ *P* < 0.01, compared to before administration.

In streptozotocin-induced untreated diabetic mice, fasting blood glucose level was significantly higher than that of untreated normal ones both before and after EEM administration (*P* < 0.01). But fasting blood glucose level did not change after single administration of EEM at doses of 2, 4, and 8 g/kg either in normal or diabetic mice (Figure 2(a)). In the diabetic mice, glibenclamide produced significant hypoglycemic effect. But glibenclamide had no effect on glucose level of normal mice.

### 3.3. Effects of EEM on Blood Glucose Level of Repeated Administration

 Changes of blood glucose level after repeated EEM and glibenclamide administration in normal and diabetic mice were shown in Figure 2(b). In normal mice, both 14 and 28 days of all repeated doses (2, 4, and 8 g/kg) of EEM and glibenclamide (0.76 mg/kg) administration caused no significant reduction in blood glucose level. Both compared to before administration and untreated diabetic mice, reduction in blood glucose level was observed after 14 days of repeated administration of 4, 8 g/kg EEM and glibenclamide to diabetic ones (*P* < 0.01). Progressive reduction was observed after 28 days of repeated 4, 8 g/kg EEM administration (*P* < 0.01). Both 14 and 28 days of 2 g/kg EEM administration resulted in no reduction in blood glucose level. Oppositely, the blood glucose level of untreated diabetic mice was continually increased during the experimental period.

### 3.4. Effects of EEM on Serum Insulin Level and ISI of Single Administration

Significant increased serum insulin level but decreased ISI were in diabetic mice (*P* < 0.01) when compared to untreated normal mice. Two hours after single administration of all doses of 2, 4, and 8 g/kg EEM and glibenclamide failed to reduce serum insulin level and to improve ISI both in normal and diabetic mice Table 1.

### 3.5. Effects of EEM on Serum Insulin Level and ISI of Repeated Administration

As showed in Table 1, in normal mice, daily administrations of 2, 4, and 8 g/kg EEM and glibenclamide over 28 days had no effect on serum insulin level and ISI. In diabetic mice, 28 days administration of 4, 8 g/kg EEM reduced serum insulin level and improved ISI when compared to untreated diabetic mice (*P* < 0.01). But 28-day administration of 2 g/kg EEM and glibenclamide had no effect on serum insulin level and ISI.

### 3.6. Effects of EEM on Serum TC and TG Levels of Single Administration

Streptozotocin injection caused significant increases in serum TC and TG levels (*P* < 0.01) when compared to untreated normal mice. Two hours after single administration of all doses of 2, 4, and 8 g/kg EEM and glibenclamide failed to reduce serum TC and TG levels both in normal and diabetic mice (Table 2).

### 3.7. Effects of EEM on Serum TC and TG Levels of Repeated Administration

Changes of serum TC and TG levels in normal and diabetic mice during 28 days of repeated EEM and glibenclamide administration were shown in Table 2. In normal mice, daily administrations of 2, 4, and 8 g/kg EEM and glibenclamide over 28 days had no effect on TC and TG levels. In diabetic mice, 28 days administration of 2 g/kg EEM and glibenclamide did not produce any significant change in TC and TG levels. However, 28 days administration of 4, 8 g/kg EEM reduced TC and TG levels when compared to untreated diabetic mice (*P* < 0.01).

### 3.8. Effects of EEM on Glycogen and TG Contents in Liver and Skeletal Muscle of Single Administration

Changes of glycogen and TG contents in liver and skeletal muscle of normal and diabetic mice after single administration of EEM and glibenclamide were shown in Table 3. In normal mice, single administrations of 2, 4, and 8 g/kg EEM and glibenclamide had no effect on glycogen and TG contents in liver and skeletal muscle. In diabetic mice, streptozotocin injection caused significant increase in TG level and decrease in glycogen level when compared to untreated normal mice (*P* < 0.01). Single administration of 2, 4, and 8 g/kg EEM and glibenclamide also did not produce any significant change in glycogen and TG contents in liver and skeletal muscle in diabetic mice.

### 3.9. Effects of EEM on Glycogen and TG Contents in Liver and Skeletal Muscle of Repeated Administration

Changes of glycogen and TG contents in liver and skeletal muscle of normal and diabetic mice during 28 days of EEM and glibenclamide administration were shown in Table 4. In normal mice, once daily administrations of 2, 4, and 8 g/kg EEM and glibenclamide for 28 days had no effect on glycogen and TG contents in liver and skeletal muscle. In diabetic mice, glycogen content was significantly lower, and TG content was significantly higher than those of untreated normal mice (*P* < 0.01). Administration of 2 g/kg EEM for 28 days did not produce any significant change in glycogen and TG contents in liver and skeletal muscle. However, repeated administration of 4, 8 g/kg EEM significantly increased glycogen content and reduced TG content in liver and skeletal muscle compared with untreated diabetic mice (*P* < 0.01). Repeated administration of glibenclamide significantly increased glycogen content (*P* < 0.01), but did not affect TG content in liver and skeletal muscle compared with untreated diabetic mice.

### 3.10. Effects of EEM on the Oral Glucose Tolerance Test

**Figure 3:**
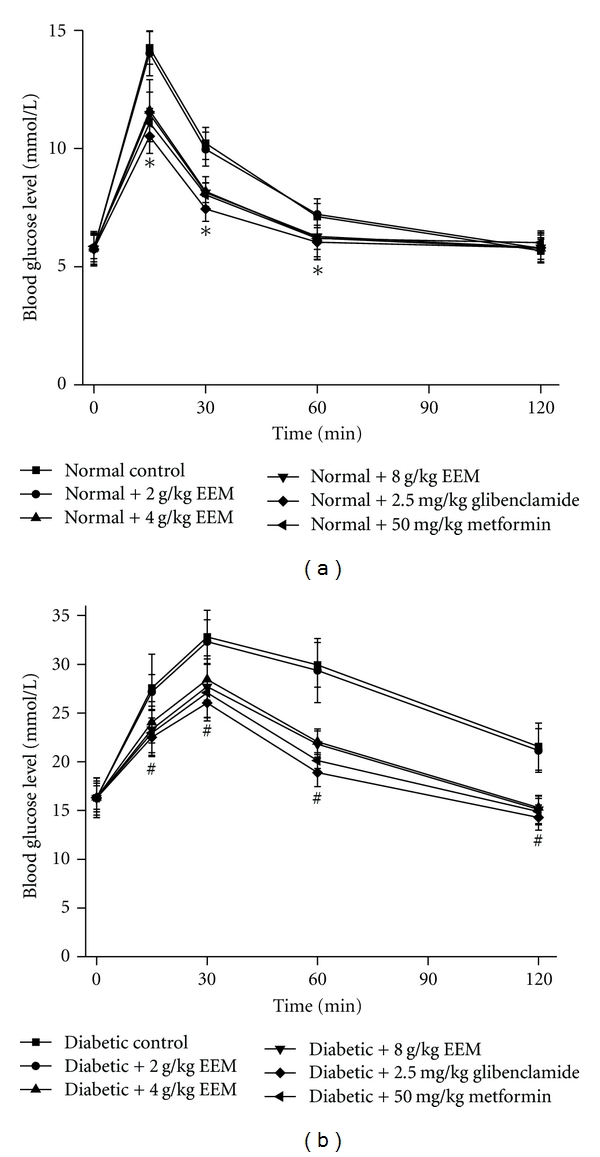
Effects of EEM on oral glucose tolerance test in normal and diabetic mice. Data are expressed as mean ± SD, *n* = 10 mice per group. *P < 0.01, compared to normal control. ^#^ *P* < 0.01, compared to diabetic control.

 In normal mice, EEM (4, 8 g/kg) significantly inhibited the increase in blood glucose level after glucose load (*P* < 0.01) (Figure 3(a)), while low dose of EEM (2 g/kg) showed no inhibitory effect. In comparative groups, both glibenclamide (2.5 mg/kg) and metformin (50 mg/kg) significantly inhibited the increase in glucose level (*P* < 0.01). In the diabetic mice, EEM (4, 8 g/kg) also showed significantly inhibitory effect on the increased blood glucose level after glucose load (*P* < 0.01) (Figure 3(b)), while low dose of EEM (2 g/kg) did not inhibit the increase in blood glucose level. The inhibitory activities of glibenclamide and metformin were observed after oral glucose load (*P* < 0.01).

When 2, 4, and 8 g/kg EEM and glibenclamide were administered for two weeks before induction of diabetes with streptozotocin and for a further 28 days afterwards, essentially similar effects were observed on blood glucose, serum insulin, ISI, serum TC, serum TG, glycogen, and TG contents in liver and skeletal muscle to those seen when EEM and glibenclamide were administered after induction of diabetes (data not shown).

## 4. Discussion

Blood glucose level was increased significantly from the 3rd day in untreated diabetic mice. This increment may be due to reduced glucose clearance apparently arising from a defect in glucose transport [24] and/or to the early appearance in insulin resistance in diabetic mice [25]. The experimental diabetic mice used in this study were type 2 diabetic ones because low dose of streptozotocin (60 mg/kg) destroyed half a population of pancreatic *β* cells and had a high blood glucose level, which are in accord with the results of previous investigations [26, 27]. Single administration EEM had no effect on blood glucose, TC, TG levels and glycogen, and TG contents in liver and skeletal muscle of both normal and diabetic mice. What is more, repeated administration EEM did not affect blood glucose, TC, TG levels and glycogen, and TG contents in liver and skeletal muscle of normal mice. These results suggest that EEM had no hypoglycemic and hypolipidemic effects on normal mice.

When studying a chronic disease such as diabetes, it is more pertinent to test the maintenance of lower blood glucose level with long-term treatment rather than the acute hypoglycemic effect after a single administration. In this study, we found that repeated administration EEM continually decreased blood glucose level in diabetic mice both after and before induction with streptozotocin. In diabetic liver and skeletal muscle, glycogen content was lower and TG content was higher than those of normal mice, which accords with other reports [28], and this phenomenon was related with impaired glucose synthesis in the mouse liver and skeletal muscle during diabetes [29]. Repeated EEM administration significantly increased tissue glycogen content in diabetic mice. In the oral glucose tolerance test, EEM (4, 8 g/kg) significantly improved hyperglycemia after oral glucose load both in normal and diabetic mice. On the other hand, the higher dose of glibenclamide (2.5 mg/kg), one of the sulfonylureas that stimulates insulin secretion [30], and metformin, one of the iguanids that inhibits hepatic glucose output [31], improved hyperglycemia after oral glucose load. Two hours after single EEM administration failed to reduce serum insulin level and to improve ISI both in normal and diabetic mice. Repeated EEM administration to diabetic mice decreased serum insulin level and improved ISI, but not for normal mice. The prevention of depletion of glycogen in the liver and skeletal muscle by EEM may be due to improved ISI, but not for stimulation of insulin release from *β* cells.

Several medicinal plants have been reported to restore activity of key enzymes of glucose and glycogen metabolism which are strongly disturbed in streptozotocin-induced diabetic mice [32, 33]. Hypoglycemic activity of EEM may arise from inhibition of hepatic glucose production. Several medicinal plants possess an insulin-sensitizing activity in experimental diabetic mice [34, 35]. Since streptozotocin-induced diabetes is accompanied by insulin resistance, EEM improved insulin sensitivity. It was reported that a traditional herbal medicine improved insulin action in streptozotocin-induced diabetic mice via enhancing insulin signaling [36, 37]. So other underlying mechanisms of the blood glucose lowering activity of EEM need further study.

The philosophy behind the medicinal uses of traditional Chinese medicine is holistic in nature, which differs from western medicines, and all chemical components present in a particular traditional Chinese medicine are believed to be of therapeutic importance. It was generally recommended to separate and measure the active chemical ingredients for standardization and quality control of herbal products. What is more, the confirmation of main constituent in herb and herbal extract helps to clarify its pharmacological effects. Previous studies reported that the *Mirabilis jalapa *L. root contains phytocompounds, such as alkaloids, glycosides, carbohydrates, and phytosterols [9, 17]. Phytochemical analysis shows that the *Mirabilis jalapa *L. roots contain plentiful trigonelline. As one of the components of *Mirabilis jalapa *L. root, trigonelline reduced blood glucose level in animal and human [19–22] and improved glucose tolerance in diabetes with obesity by oral glucose tolerance test. Trigonelline also reduced TC, free plasma cholesterol, and TG levels in the serum, liver, and adipose tissue [38, 39]. What is more, trigonelline decreased serum insulin, serum tumor necrosis factor *α* levels, and liver fatty acid synthase activity and increased carnitine palmitoyl transferase, glucokinase activities, and glucokinase/glucose-6-phosphatase ratio in the liver [38, 40]. Therefore, it was reasonable to suggest that the insulin sensitivity, hypoglycemia, and hypolipidemia profile of EEM might be connected to the existence of the phytocompounds, especially the trigonelline. Meanwhile, our results clearly demonstrate that administration EEM provides beneficial insulin sensitivity, hypoglycemic, and hypolipidemic effects especially on diabetic mice both after and before induction with streptozotocin.

In conclusion, our study demonstrates that *Mirabilis jalapa* L. root can be used to treat type 2 diabetes with hyperlipidemia. *Mirabilis jalapa* L. root may be developed as an oral hypoglycemic agent or functional food for diabetic patients with hyperlipidemia and for persons with high risk of diabetes. This finding represents an experimental confirmation of the Chinese traditional use of this plant for diabetic treatment. Consequently, consumption of *Mirabilis jalapa* L. root may prevent the complication of hyperglycemia associated with diabetes. Finally, the precise mechanism(s) and site(s) of this activity and the active constituent(s) of *Mirabilis jalapa* L. root still need to be determined in addition to toxicological studies in further experiments.

## Figures and Tables

**Table 1:** Effects of single and repeated EEM administration on serum insulin level and ISI in normal and diabetic mice.

Group	Single administration		Repeated administration	
	Serum insulin (mU/L)	ISI	Serum insulin (mU/L)	ISI
Normal control	11.80 ± 0.83	−1.88 ± 0.06	11.49 ± 1.17	−1.85 ± 0.04
Normal + 2 g/kg EEM	11.65 ± 0.44	−1.88 ± 0.04	11.68 ± 0.60	−1.87 ± 0.04
Normal + 4 g/kg EEM	11.60 ± 0.79	−1.86 ± 0.06	11.55 ± 0.63	−1.86 ± 0.05
Normal + 8 g/kg EEM	11.06 ± 0.30	−1.86 ± 0.05	11.67 ± 0.64	−1.86 ± 0.05
Normal + glibenclamide	11.91 ± 0.36	−1.89 ± 0.04	11.54 ± 0.75	−1.85 ± 0.06
Diabetic control	16.74 ± 0.53*	−2.45 ± 0.05*	14.81 ± 1.04*	−2.48 ± 0.05*
Diabetic + 2 g/kg EEM	16.43 ± 0.43	−2.44 ± 0.05	14.65 ± 1.88	−2.48 ± 0.08
Diabetic + 4 g/kg EEM	16.01 ± 0.53	−2.43 ± 0.03	11.88 ± 0.90	−2.04 ± 0.08^#^
Diabetic + 8 g/kg EEM	16.07 ± 0.95	−2.42 ± 0.04	11.68 ± 0.81	−2.03 ± 0.05^ #^
Diabetic + glibenclamide	36.83 ± 4.82^#^	−2.40 ± 0.09	39.15 ± 6.55^#^	−2.42 ± 0.09

Insulin sensitivity index (ISI) = log (1/fasting plasma glucose × serum insulin). Data are expressed as mean ± SD, *n* = 10 mice per group. *P < 0.01 when compared to normal control. ^#^ *P* < 0.01 when compared to diabetic control.

**Table 2:** Effects of single and repeated EEM administration on serum TC and TG levels in normal and diabetic mice.

Group	Single administration		Repeated administration	
	TC (mmol/L)	TG (mmol/L)	TC (mmol/L)	TG (mmol/L)
Normal control	2.13 ± 0.20	0.74 ± 0.07	2.13 ± 0.16	0.75 ± 0.06
Normal + 2 g/kg EEM	2.11 ± 0.20	0.75 ± 0.11	2.14 ± 0.17	0.75 ± 0.06
Normal + 4 g/kg EEM	2.17 ± 0.22	0.77 ± 0.09	2.14 ± 0.20	0.74 ± 0.05
Normal + 8 g/kg EEM	2.08 ± 0.22	0.75 ± 0.08	2.10 ± 0.16	0.73 ± 0.06
Normal + glibenclamide	2.11 ± 0.21	0.75 ± 0.08	2.11 ± 0.14	0.74 ± 0.08
Diabetic control	3.47 ± 0.33*	1.26 ± 0.12*	3.49 ± 0.32*	1.28 ± 0.09*
Diabetic + 2 g/kg EEM	3.45 ± 0.39	1.24 ± 0.10	3.47 ± 0.26	1.24 ± 0.11
Diabetic + 4 g/kg EEM	3.44 ± 0.43	1.24 ± 0.12	2.92 ± 0.22^#^	1.01 ± 0.11^#^
Diabetic + 8 g/kg EEM	3.44 ± 0.31	1.25 ± 0.10	2.82 ± 0.25^#^	0.97 ± 0.07^#^
Diabetic + glibenclamide	3.48 ± 0.34	1.23 ± 0.10	3.46 ± 0.13	1.27 ± 0.09

Data are expressed as mean ± SD, *n* = 10 mice per group. *P < 0.01 when compared to normal control. ^#^ *P* < 0.01 when compared to diabetic control.

**Table 3:** Effects of single EEM administration on glycogen and TG contents in liver and skeletal muscle of normal and diabetic mice.

Group	Liver		Skeletal muscle	
	Glycogen (mg/g)	TG (*μ*mol/g)	Glycogen (mg/g)	TG (*μ*mol/g)
Normal control	12.73 ± 0.89	3.40 ± 0.22	1.69 ± 0.16	0.116 ± 0.005
Normal + 2 g/kg EEM	12.58 ± 0.91	3.40 ± 0.20	1.67 ± 0.12	0.115 ± 0.005
Normal + 4 g/kg EEM	12.46 ± 0.74	3.47 ± 0.29	1.65 ± 0.06	0.117 ± 0.006
Normal + 8 g/kg EEM	12.57 ± 0.96	3.44 ± 0.31	1.69 ± 0.12	0.115 ± 0.007
Normal + glibenclamide	12.66 ± 0.96	3.40 ± 0.35	1.68 ± 0.15	0.111 ± 0.010
Diabetic control	6.77 ± 0.55*	5.17 ± 0.26*	2.89 ± 0.20*	0.215 ± 0.017*
Diabetic + 2 g/kg EEM	6.99 ± 0.60	5.27 ± 0.48	2.89 ± 0.21	0.213 ± 0.016
Diabetic + 4 g/kg EEM	7.04 ± 0.55	5.35 ± 0.39	2.72 ± 0.21	0.219 ± 0.013
Diabetic + 8 g/kg EEM	7.20 ± 0.35	5.28 ± 0.40	2.73 ± 0.36	0.201 ± 0.014
Diabetic + glibenclamide	7.18 ± 0.56	5.25 ± 0.35	2.76 ± 0.28	0.199 ± 0.013

Data are expressed as mean ± SD, *n* = 10 mice per group. *P < 0.01 when compared to normal control.

**Table 4:** Effects of repeated EEM administration on glycogen and TG contents in liver and skeletal muscle of normal and diabetic mice.

Group	Liver		Skeletal muscle	
	Glycogen (mg/g)	TG (*μ*mol/g)	Glycogen (mg/g)	TG (*μ*mol/g)
Normal control	12.43 ± 1.19	3.39 ± 0.28	1.66 ± 0.16	0.126 ± 0.009
Normal + 2 g/kg EEM	12.36 ± 1.05	3.45 ± 0.16	1.55 ± 0.14	0.123 ± 0.007
Normal + 4 g/kg EEM	12.67 ± 1.00	3.50 ± 0.25	1.72 ± 0.17	0.131 ± 0.011
Normal + 8 g/kg EEM	12.75 ± 1.33	3.35 ± 0.27	1.70 ± 0.15	0.131 ± 0.008
Normal + glibenclamide	12.81 ± 1.06	3.28 ± 0.26	1.71 ± 0.12	0.128 ± 0.007
Diabetic control	6.86 ± 0.41*	5.28 ± 0.21*	2.75 ± 0.20*	0.226 ± 0.016*
Diabetic + 2 g/kg EEM	7.28 ± 0.36	5.29 ± 0.34	2.75 ± 0.28	0.215 ± 0.019
Diabetic + 4 g/kg EEM	9.38 ± 1.14^#^	4.24 ± 0.37^#^	2.04 ± 0.22^#^	0.170 ± 0.008^#^
Diabetic + 8 g/kg EEM	9.79 ± 0.99^#^	4.09 ± 0.30^#^	2.03 ± 0.17^#^	0.164 ± 0.011^#^
Diabetic + glibenclamide	10.81 ± 0.77^#^	5.15 ± 0.33	1.79 ± 0.13^ #^	0.221 ± 0.020

Data are expressed as mean ± SD, *n* = 10 mice per group. *P < 0.01 when compared to normal control. ^#^ *P* < 0.01 when compared to diabetic control.
